# Launching genomics into the cloud: deployment of *Mercury*, a next generation sequence analysis pipeline

**DOI:** 10.1186/1471-2105-15-30

**Published:** 2014-01-29

**Authors:** Jeffrey G Reid, Andrew Carroll, Narayanan Veeraraghavan, Mahmoud Dahdouli, Andreas Sundquist, Adam English, Matthew Bainbridge, Simon White, William Salerno, Christian Buhay, Fuli Yu, Donna Muzny, Richard Daly, Geoff Duyk, Richard A Gibbs, Eric Boerwinkle

**Affiliations:** 1 Human Genome Sequencing Center, Baylor College of Medicine, One Baylor Plaza, Houston, TX 77030, USA; 2 DNAnexus, Mountain View, CA 94040, USA; 3 Department of Molecular and Human Genetics, Baylor College of Medicine, Houston, TX 77030, USA; 4 Human Genetics Center, University of Texas Health Science Center at Houston, Houston, TX 77030, USA

**Keywords:** NGS data, Variant calling, Annotation, Clinical sequencing, Cloud computing

## Abstract

**Background:**

Massively parallel DNA sequencing generates staggering amounts of data. Decreasing cost, increasing throughput, and improved annotation have expanded the diversity of genomics applications in research and clinical practice. This expanding scale creates analytical challenges: accommodating peak compute demand, coordinating secure access for multiple analysts, and sharing validated tools and results.

**Results:**

To address these challenges, we have developed the *Mercury* analysis pipeline and deployed it in local hardware and the Amazon Web Services cloud via the DNAnexus platform. *Mercury* is an automated, flexible, and extensible analysis workflow that provides accurate and reproducible genomic results at scales ranging from individuals to large cohorts.

**Conclusions:**

By taking advantage of cloud computing and with *Mercury* implemented on the DNAnexus platform, we have demonstrated a powerful combination of a robust and fully validated software pipeline and a scalable computational resource that, to date, we have applied to more than 10,000 whole genome and whole exome samples.

## Background

Whole exome capture sequencing (WES) and whole genome sequencing (WGS) using next generation sequencing (NGS) technologies [1] have emerged as compelling paradigms for routine clinical diagnosis, genetic risk prediction, and patient management [2]. Large numbers of laboratories and hospitals routinely generate terabytes of NGS data, shifting the bottleneck in clinical genetics from DNA sequence production to DNA sequence analysis. Such analysis is most prevalent in three common settings: first, in a clinical diagnostics laboratory (e.g. Baylor’s Whole Genome Laboratory http://www.bcm.edu/geneticlabs/) testing single patients or families with presumed heritable disease; second, in a cancer-analysis setting where the unit of interest is either a normal-tumor tissue pair or normal-primary tumor-recurrence trio [3]; and third, in biomedical research studies sequencing a sample of well-phenotyped individuals. In each case, the input is a DNA sample of appropriate quality having a unique identification number, appropriate informed consent, and relevant clinical and phenotypic information.

As these new samples are sequenced, the resulting data is most effectively examined in the context of petabases of existing DNA sequence and the associated meta-data. Such large-scale comparative genomics requires new sequence data to be robustly characterized, consistently reproducible, and easily shared among large collaborations in a secure manner. And while data-management and information technologies have adapted to the processing and storage requirements of emerging sequencing technologies (e.g., the CRAM specification [4]), it is less certain that appropriate informative software interfaces will be made available to the genomics and clinical genetics communities. One element bridging the technology gap between the sequencing instrument and the scientist or clinician is a validated data processing pipeline that takes raw sequencing reads and produces an annotated personal genome ready for further analysis and clinical interpretation.

To address this need, we have designed and implemented *Mercury*, an automated approach that integrates multiple sequence analysis components across many computational steps, from obtaining patient samples to providing a fully annotated list of variant sites for clinical applications. *Mercury* fully integrates new software with existing routines (e.g., Altas2 [5]) and provides the flexibility necessary to respond to changing sequencing technologies and the rapidly increasing volume of relevant data. *Mercury* has been implemented on both local infrastructure and in a cloud computing platform provided by DNAnexus using Amazon Web Services (AWS). While there are other NGS analysis pipelines, some of which have even been implemented in the cloud [6], the combination of *Mercury* and DNAnexus together provide for the first time a fully integrated genomic analysis resource that can serve the full spectrum of users.

## Results and discussion

**Figure 1:**
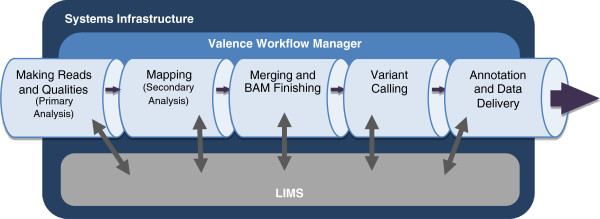
***Mercury *Data Flow.** 1) Sequencing Instrument raw data is passed to vendor primary analysis software to generate sequence reads and base call confidence values (qualities). 2) Reads and qualities are passed to a mapping tool (BWA) for comparison to a reference genome to determine the placement of reads on the reference (producing a BAM file). 3) Individual sequence event BAMs are merged to make a single sample-level BAM file that then is processed in preparation for variant calling. 4) Atlas-SNP and Atlas-indel are used to identify variants and produce variant files (VCF). 5) Annotation adds biological and functional information to the variant lists and formats them for delivery.

Figure 1 provides an overview of the *Mercury* data processing pipeline. Source information includes sample and project management data and the characteristics of library preparation and sequencing. This information enters the pipeline either directly from the user or from a laboratory information management system (LIMS). The first step, generating sequencing reads, is based on the vendor’s primary analysis software, which generates sequence reads and base-call confidence values (qualities). The second step maps the reads and qualities to the reference genome using a standard mapping tool, such as BWA [7,8], producing a BAM [9] (binary alignment/map) file. The third step produces a “finished” BAM that includes sorting, duplicate marking, indel realignment, base quality recalibration, and indexing (using a combination of tools including SAMtools [9], Picard (http://picard.sourceforge.net), and GATK [10]). The fourth step in *Mercury* uses the Atlas2 suite [5,11] (Atlas-SNP and Atlas-indel) to call variants and produce a variant file (VCF). The fifth step adds biological and functional annotation and formats the variant lists for delivery. Each step is described in detail in the Methods section, as is the flow of information between steps.

*Mercury* has been optimized for the Illumina HiSeq (Illumina, Inc.; San Diego, CA) platform, but the generalized workflow framework is adaptable to other settings. The entire pipeline has been implemented both locally and in a cloud computing environment. All relevant code and documentation are freely available online (https://www.hgsc.bcm.edu/content/mercury) and the scalable cloud solution is available within the DNAnexus library (http://www.dnanexus.com/). Sensible default parameters have already been determined so that researchers and clinicians can reliably analyze their data with *Mercury* without needing to configure any of the constituent programs or obtaining access to large computational resources, and they can do so in a manner compliant with multiple regulatory frameworks.

### Local workflow management

Implementing a robust analysis framework that incorporates a heterogeneous collection of software tools presents many challenges. Running disparate software modules with varying inputs and outputs that depend on each other’s results requires appropriate error checking and centralized output logging. We therefore developed a simple yet extensible workflow management framework, *Valence* (http://sourceforge.net/projects/valence/), that manages the various steps and dependencies within *Mercury* and handles internal and external pipeline communication. This formal approach to workflow management helps maximize computational resource utilization and seamlessly guides the data from the sequencing instrument to an annotated variant file ready for clinical interpretation and downstream analysis.

*Valence* parses and executes an analysis protocol described in XML format with each step treated as either an action or a procedure. An action is defined as a direct call to the system to submit a program or script to the job scheduler for execution; a procedure is defined a collection of actions, which is itself a workflow. This design allows the user to easily add, remove, and modify the steps of any analysis protocol. A protocol description for a specific step must include the required cluster resources, any dependencies on other steps, and a command element that describes how to execute the program or script. To ensure that the XML wrappers are applicable to any run, the command is treated as a string template that allows XML variables to be substituted into the command prior to execution. Thus, a single XML wrapper describing how to run a program can be applied to different inputs. *Valence* can be deployed on any cluster with a job scheduler (e.g., PBS, LSF, SGE), implementing a database to track both the job (the collection of all the steps in a protocol to be executed) and the status (“Not Started,” “Running,” “Finished,” “Failed”) of any action associated with the job.

*Mercury* users can easily incorporate new analysis tools into an existing pipeline. For example, we recently expanded the scope of our pipeline to include Tiresias (https://sourceforge.net/projects/tiresias/), a structural variant caller focused on mobile elements, and ExCID (https://github.com/cbuhay/ExCID), an exome coverage analysis tool designed to provide clinical reports on under-covered regions. To incorporate Tiresias and ExCID into the *Mercury* pipeline, we needed only to specify the compute requirements and add the appropriate command to the existing XML workflow definition; *Valence* then automatically handles all data passing, logging, and error reporting.

### Cloud workflow management

*Mercury* has been instantiated in the cloud via the DNAnexus platform (utilizing AWS’s EC2 and S3). DNAnexus provides a layer of abstraction that simplifies development, execution, monitoring, and collaboration on a cloud infrastructure. The constituent steps of the *Mercury* pipeline take the form of discrete “applets,” which are then linked to form a workflow within the DNAnexus platform infrastructure. Using the workflow construction GUI, one can add applets (representing each step) to the workflow and create a dependency graph by linking the inputs and outputs of subsequent applets. Inputs are provided to an instance of the workflow, and the entire workflow is run within the cloud infrastructure. The various steps within the workflow are then executed based on the dependency graph. As with *Valence*, individual applets can be configured to run with a specific set of computational resource requirements such as memory, local disk space, and number of cores and processors. We are currently working to merge the local and cloud infrastructure elements by integrating the upload agent into *Valence*, allowing *Valence* to trigger a DNAnexus workflow once all the data is successfully uploaded. Such coordination would serve to transparently support analysis bursts.

The *Mercury* pipeline within DNAnexus comprises code that uses the DNAnexus command-line interface to instantiate the pipeline in the cloud. The *Mercury* code for DNAnexus is executed on a local terminal. For example, one may provide a list of sample FASTQ files and sample meta-data locations to *Mercury*, at which point *Mercury* uploads the data and instantiates the predefined workflow within DNAnexus. On average, on a 100 Mbps connection, we were able to upload at a rate of ~14 MB/sec. We were able to parallelize this uploading process, yielding an effective upload rate of ~90 MB/sec. The size of a typical FASTQ file from WES with 150X coverage has a compressed (bzip2) file size of approximately 3 GB. Uploading such a file from a local server took less than five minutes. After sample data are uploaded to the DNAnexus environment, the workflow is instantiated in the cloud.

**Figure 2:**
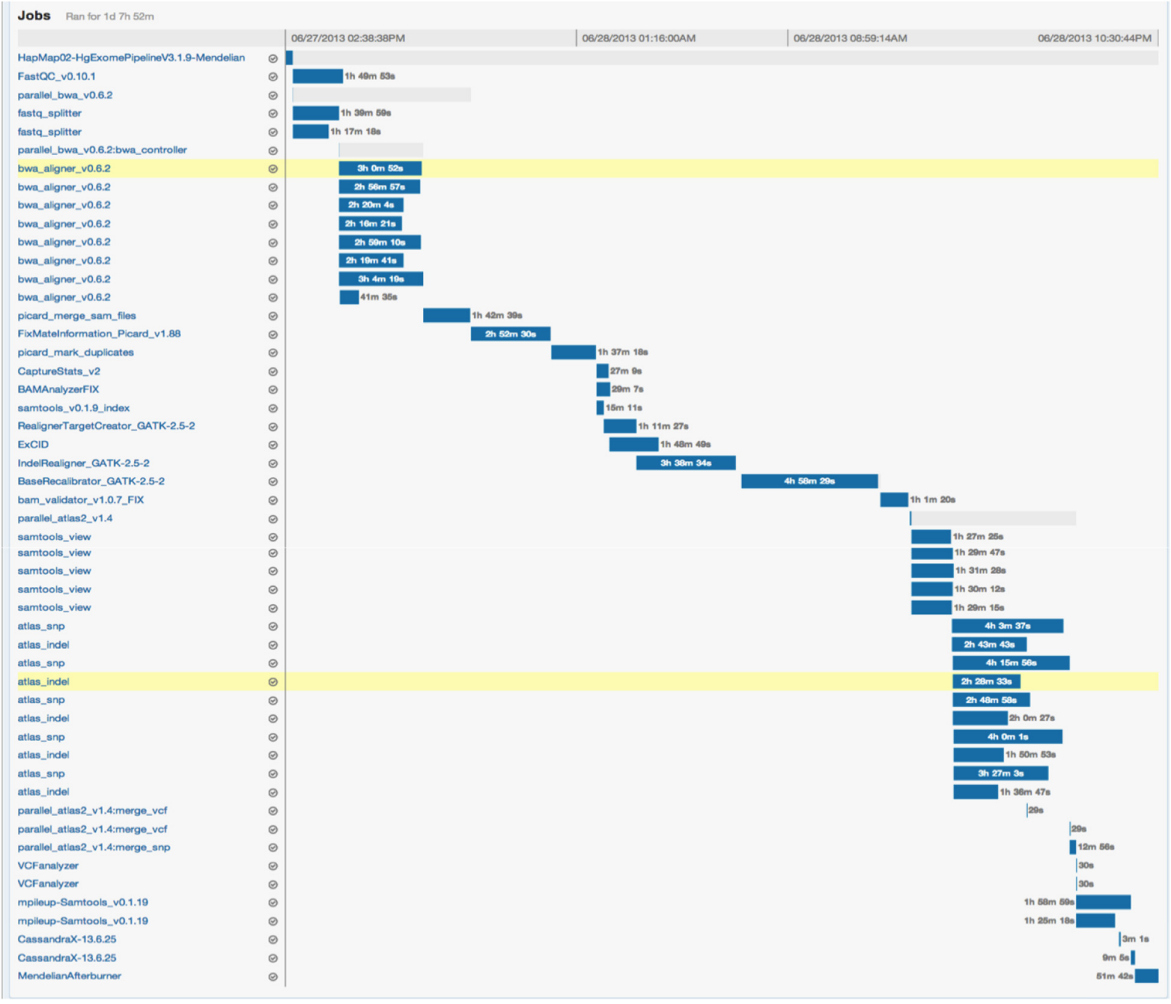
**Workflow monitoring in DNAnexus.** The GUI for applet monitoring displays the progress as a Gantt chart. The left panel lists the various steps including the parallelization steps with each row corresponding to a compute instance. A particular step can be clicked to determine the exact inputs and output or logs of execution for that step. Here we show a snapshot of the webpage displaying the progress of execution for the NA12878 exome analysis.

Progress can be monitored online using the DNAnexus GUI (Figure 2) or locally through the *Mercury* monitoring code. To achieve full automation, the monitoring code can be made a part of a local process to poll for analysis status at regular intervals and start analysis of new sequences automatically upon completion of sequencing. When the *Mercury* monitoring code detects successful completion of analysis an email notification is sent out. The results can either be downloaded to the local server or the user can view various tracks and data with a native genome browser.

### Performance

Turn-around time for raw data generation on most NGS platforms is already considered long for many clinical applications, so minimizing analysis time is a primary goal of the *Mercury* pipeline. By maintaining a high-performance computing cluster consisting of hundreds of 8-core, 48 GB RAM nodes and introducing *Mercury* into the sequencing pipeline, we can minimize wait times by ensuring that compute resources are always available for all sequence events as the instrument produces the data. To match compute resources to production requirements, we carefully monitor the run times (and RAM and CPU requirements) of each step in the *Mercury* pipeline. Table 1 describes the pipeline for each compute-intensive step, from the generation of reads and qualities from raw data (bcl to FASTQ) to generation of post-processed annotated variant files (VCFs). Resource requirements for each step are given in terms of fraction of an 8-core node (CPU) and RAM allocated. Note that some steps under-utilize available CPUs because they require most (or all) of the RAM available on a given node. For data generated via WES from human samples, the *Mercury* pipeline requires less than 36 hours of wall-clock time and 15 node-hours (i.e., the equivalent of one whole node fully dedicated to processing for 15 hours). Run times and resource requirements will vary with data type, reference genome, and computing hardware configuration.

**Table 1:** *Mercury* computational resource requirements

	**Data gen**	**Alignment**		**BAM finishing**						**Variants**		**Anno**
	**BCL to FastQ**	**BWA align**	**BWA sample**	**Mates, Dupe, Stats**	**Cap & Cvrg Metrics**	**GATK indel targets**	**GATK indel realign**	**GATK recal**	**BAM valid**	**Atlas SNP**	**Atlas Indel**	**Cassandra**
Nodes	1	1	0.333	0.5	0.125	1	0.333	1	0.125	0.167	0.167	0.167
RAM	48	48	15	28	3	48	14	32	4	7	7	8
Hours	3.62	1.84	1.38	3.39	1.30	0.28	2.25	3.04	0.75	9.00	7.51	1.71
Node*hrs	3.62	1.84	0.46	1.70	0.16	0.28	0.75	3.04	0.09	1.50	1.25	0.29

All estimates are approximate for whole exome and light-skim whole genome (~10-20 Gbp of data) sequenced on Illumina HiSeq and processed with the most recent versions of RTA and Casava. Nodes are 8-core, 48 GB RAM, with ~3 GHz Intel CPUs and ~1 TB of local scratch disk. Steps include all aspects of the pipeline from building reads and qualities (fastQ) from raw data (bcl files), through alignment and BAM generation using the BWA aligner, and BAM finishing with GATK post-processing and duplicate marking, capture and coverage metric calculation, and BAM file validation, finally producing variants from the Atlas2 variant calling suite with annotations from our annotator, Cassandra.

After porting each element of the *Mercury* pipeline into the DNAnexus environment, the tools (i.e., “apps”) can be run on the cloud in environments with different CPU, RAM, disk, and bandwidth resources to optimize wall-clock time and cost-efficiency. Parallelization within a pipeline reduces the time for a single run, which is useful for quick development cycles or time-sensitive applications. In addition, many parallel pipelines can be run simultaneously. The current peak usage for the *Mercury* pipeline on DNAnexus is approximately 12,000 cores. This throughput is a small fraction of the theoretical maximum that could be achieved in AWS. For the standard implementation of *Mercury* in the cloud analyzing a validation exome (Hapmap sample NA12878), the total wall time to produce an annotated variant call VCF (starting with paired-end FASTQ files) is approximately one day.

### Data management, sharing, reliability, and compliance

Large projects present a large data management challenge. For example, the Baylor-Hopkins Center for Mendelian Genomics has generated WES data from approximately 2,000 samples and the Human Genome Sequencing Center at Baylor College of Medicine has generated more than 10,000 WES and 3,500 WGS data from research samples from the CHARGE consortium [12]. As a pilot study, we processed 1,000 WES data sets–approximately 80 terabytes of genomic data–from the Center for Mendelian Genomics using *Mercury* in DNAnexus. Using multi-threaded uploads we were able to deliver data into the cloud at an average rate of ~960 exomes per day. Once uploaded, the data is analyzed with *Mercury*, and the resulting variants can be accessed for further analysis via a web GUI. Data can also be tagged, and these tags can be filtered or retrieved. Runs of individual pipelines and tools can be queried in a similar way.

As datasets become larger, multi-site collaborative consortiums play an increasingly important role in contemporary biomedical research. A major advantage of cloud computing over local computing is that cloud storage can be shared across multiple organizations. Instead of each collaborator maintaining a local copy of the data and working in isolation, cloud users can be given appropriate access permissions so some researchers can view and download the results, others can run analyses on the data and build tools, and those with administrative privileges can determine access to the project. This data paradigm is the only feasible approach to giving patients meaningful access to their own genomic data.

### Comparison to existing methods

A number of other tools and services provide similar functionality to *Mercury* on DNAnexus, with differing approaches to extensibility, ease of use for non-programmers, support for local or cloud infrastructures, and software available by default (Table 2). For example, the academic service *Galaxy* primarily focuses on extensibility and building a developer community [13-15]. *Seven Bridges* is a commercial service that combines a few fixed pipelines with a visually distinctive workflow editor. *Chipster* is an academic service that packages a variety of NGS tools in addition to a variety of microarray tools and combines these with visualization of data summaries and QC metrics [16]. *Anduril* is designed to manage a local cluster and contains packages for a variety of tasks, including alignment and variant calling as well as image analysis and flow cytometry, which are not addressed by the other cloud services surveyed [17]. With respect to the software used in sequence production pipelines, *Mercury* is most distinguished by its Atlas variant caller and the extensive annotations provided by its Cassandra annotation tool (https://www.hgsc.bcm.edu/software/cassandra).

**Table 2:** A feature summary of Mercury in DNAnexus and similar tools and services

	**Mercury in DNAnexus**	**Galaxy**	**Seven bridges**	**Chipster**	**Anduril**
Mapper	BWA	No canonical (many)	BWA	BWA	BWA/Bowtie
Variant caller	Atlas2	No canonical (many)	GATK	Samtools mpileup	Samtools/GATK, Varscan
Annotation	Cassandra	snpEff, AlleleFrequency	snpEff, ANNOVAR	Bioconductor	ANNOVAR
Visualization	Built-in browser	Built-in browser	Links to IGV	Built-in browser	No
Runs on local hardware	No*	Yes	No	Yes	Yes
Runs on cloud infrastructure	Yes	Yes	Yes	Jobs queued to public server	No
HIPAA compliant	Yes	No	No	No	Not Applicable (local only)
Requires setup configuration	No	Yes	No	Yes	Yes
Can add tools independently	Yes	Yes	By request	No	No
Data sharing & collaboration	Yes	Yes	No	No	No

*Mercury can be run on local hardware via Valence, but does not include all of the security and data sharing features of Mercury in DNAnexus.

### Future directions

As genomic studies transition from extensive us of whole exome capture sequencing methods to an emphasis on whole genome sequencing [18], we can take advantage of *Mercury*’s flexibility to adapt to shifting research priorities. Currently, the sequencing community uses whole exome sequencing because of its comparatively low cost and because it enriches for biological signals that are readily interpretable. However, with changing price structures, the advantages of eliminating the capture step in the laboratory, improvements in lossless compression of sequence data, and improved annotation of the non-protein coding region of the genome (e.g., genome.ucsc.edu/ENCODE/), whole genome sequencing may become a more appealing option. Early adopters of whole genome sequencing will certainly include medical sequencing for research and diagnostic purposes. Therefore, a high priority for *Mercury* is the continual updates to the annotation information using web-based databases. Such web-based annotation updates can incorporate the latest developments related to genome function in near real-time.

Clinically relevant annotation is of particular interest in the growing area of cancer somatic mutation. Currently, disease-related annotation considers inherited disease alleles gleaned from OMIM (http://www.omim.org), HGMD (http://www.hgmd.org; assuming the user has the appropriate license to use this data), and genome-wide association studies (http://www.genome.gov/gwastudies). Similarly informative databases, such as the Catalogue of Somatic Mutations in Cancer (COSMIC; http://www.sanger.ac.uk/genetics/CGP/cosmic/), are emerging and will need to be fully vetted and incorporated into Cassandra. The goal of *Mercury* is to provide a simple solution for end-to-end sequence analysis so that non-expert users can obtain a list of annotated and prioritized variants as rapidly as possible, and so that expert users can augment and modify the pipeline to meet specialized needs.

## Conclusions

The long-anticipated NGS data deluge [19] has now arrived. The first personal NGS genome was published in 2007 [4], and today we estimate that the number of available exomes and genomes approaches one hundred thousand. It is equally impressive that the application of NGS to biomedical research and clinical medicine is rapidly becoming standard. Such applications are driven by the utility of sequence data, as demonstrated by a number of instances where DNA sequencing has been used not only for diagnostic purposes but also to reveal more efficacious therapies [18].

By taking advantage of cloud computing and with *Mercury* implemented on the DNAnexus platform, we have demonstrated a powerful combination of a robust and fully validated software pipeline and a scalable computational resource. To date, we have analyzed thousands of samples (using the AWS cloud: EC2 and S3), including a population study comprising 3,500 samples and 10,000 samples for which WGS and WES were generated, respectively, more than 1,000 Mendelian disease cases shared with data consumers all over the world, and smaller projects such as 50 exome trios and 30 cancer WGS tumor/normal pairs. To our knowledge, these projects represent the largest genomic analysis effort to be realized in the cloud to date. They presage a wave of genomic computing that will transform how genomic data is analyzed and delivered to the scientific community, into clinical practice, and eventually directly into the hands of patients and advocates.

## Methods

### Reads and read qualities

Making reads and assigning those reads a quality metric is the only step of *Mercury* that relies almost exclusively upon vendor-provided software. Processing raw data files down to signal intensities and then taking the signal data through the demultiplexing (i.e., assigning a sequence read to an individual barcoded sample), base calling, and quality scoring processes is integrally tied to the sequencing chemistry and machine instrumentation. Vendors, in collaboration with the user community, are constantly developing and improving this primary data input step. As such, the integrated *Mercury* pipeline is triggered by the completion of the data transfer from the instrument into the local compute environment, at which point vendor tools are used to generate a FASTQ file containing reads and qualities for each sequence event. The current version of *Mercury* is optimized for the Illumina HiSeq instrument, so it currently integrates with Bcl2FastQ (http://support.illumina.com/downloads/bcl2fastq_conversion_software_184.ilmn), the Illumina analysis toolkit. Modularity of the *Mercury* pipeline facilitates incorporation of upgrades to Bcl2FastQ and the replacement of Bcl2FastQ with functionally similar tools as necessary. At the end of this step, data from a flow cell is broken down into individual sequence events associated with each barcode.

### Mapping and BAM Generation

Once reads and qualities are generated, each sequence event is mapped to a reference genome. The Burrows-Wheeler Aligner (BWA) [8] is the current preferred mapping tool for Illumina HiSeq data, though *Mercury*’s modular design allows for alternative mapping tools. The mapping process for short reads consists of a “seeding” step that identifies regions of similarity between the read and reference, and then a local alignment stage that makes comparisons among reads in a region. Through this iterative process, BWA identifies the most likely placement for the reads and the differences between each read and the reference.

Once mapping is complete, additional processing steps are taken to improve the resulting BAM files. The data is sorted by mapping position, duplicate reads (artifacts of the sample preparation process) are flagged, and metadata summarizing data production information is added. Further downstream, base quality recalibrations and local realignment around regions with indel calls are performed to further improve the information content of variant calls. For cases in which data from a single sample spans multiple sequence events, *Mercury* provides the means to merge individual sequence event BAMs into a single sample-level BAM that can then be used for downstream analysis.

### Variant calling

*Mercury* is designed to provide robust variant calling for readily available whole exome sequencing data but is extensible to similar functionality for whole genome data. We have implemented Atlas2 [5,11], an SNV- and indel-calling suite. Atlas2 uses a logistic regression approach to detect systematic sequencing errors using context-sensitive input variables, following three hierarchical steps: a logistic regression model to characterize systematic sequencing errors, a probability score that detects and removes errors, and a minimal heuristics filters as *post-hoc* quality assurances to address mapping and capture biases. Atlas2 has high SNP-discovery sensitivity and specificity when applied to the 1000 Genomes Whole Exome data [11]. The accuracy of homozygous and heterozygous SNPs are as high as 99.8% and 99.6%, respectively, when compared to the SNP array data. We have also demonstrated high quality in short indel discoveries [5].

### Technical validation and reproducibility

To enable clinical applications, we characterized *Mercury*’s reproducibility of calls in gene-coding regions. We generated VCFs from two independent samples and compared the high quality variant calls from one to the variant calls of any quality from the other sample. The process was then repeated with the sample roles reversed. At the end of this process, we produced three sets of comparisons: SNPs with passing evidence in both samples, SNPs that only have passing evidence in the first sample, and those that only have passing evidence in the second sample. For illustrative purposes, reproducibility through technical replicates for eight samples meeting the basic criteria of greater than 90% of targeted bases covered at 20x or better is shown in Table 3. These observations indicate strong consistency (less than 2% variability between technical replicates), with approximately 24,000 SNPs found consistently in the overlap.

**Table 3:** **Technical replicate data for the *Mercury *pipeline for eight samples sequenced in duplicate (A and B)**

**Sample #**	**A only**	**A only (%)**	**A and B**	**A and B (%)**	**B only**	**B only (%)**
HS-1011	133	0.561%	23,320	98.409%	244	1.029%
HS-1015	155	0.542%	28,312	98.910%	157	0.548%
HS-1016	155	0.644%	23,752	98.732%	150	0.624%
HS-1017	105	0.456%	22,767	98.768%	179	0.777%
HS-1018	165	0.693%	23,531	98.795%	122	0.512%
HS-1019	162	0.682%	23,441	98.686%	150	0.631%
HS-1020	493	2.041%	23,518	97.355%	146	0.604%
HS-1021	161	0.681%	23,188	98.125%	282	1.193%
Average	191	0.718%	23,979	98.473%	179	0.613%

We then assessed *Mercury’s* accuracy by evaluating the sensitivity and specificity of the *Mercury* SNP calls. SNP array data and whole-exome sequencing data were generated from a single sample, and seven exome technical replicates were analyzed with *Mercury*. The array data indicated the presence of 1,927 SNPs and 2,814 homozygous reference sites that overlapped with regions targeted by the exome capture reagent. Table 4 details *Mercury*’s ability to recover these sites, averaging almost 99% sensitivity across the replicates. Moreover, *Mercury* made no SNP calls in any of the replicates for any of the SNP array homozygous reference sites, and achieved sufficient coverage to consider those sites reference homozygous.

**Table 4:** **Concordance of SNP array and *Mercury *data**

**Replicate**	**PASS**	**ALL**
1	1897	1906
2	1900	1906
3	1898	1904
4	1895	1905
5	1899	1907
6	1904	1907
7	1902	1905
Average	1899.28	1905.71
Average %	98.56	98.89

For each exome technical replicate, we report the number of passing SNPs found by *Mercury* and the total number of SNPs (passing and not passing) in the VCF that overlap the SNP array design. The SNP array contains 1,927 sites that overlap the exome capture region.

To facilitate validation by others of local installations of *Mercury* or modified applications in the cloud, we are making available the FASTQ, BAMs and VCFs of a single individual with a known Mendelian condition [20]. These data are available at http://www.ncbi.nlm.nih.gov/sra?term=SRP023104.

### Annotation

*Mercury* provides variant annotation via the Cassandra annotation suite, which describes the quality and predicted functional consequences of genomic variants, providing the biological and clinical contexts needed to assess the significance of each variant. Variants are presented to the user with all quality control metrics produced by Atlas2, the “pileup string,” [9] and the theoretical mappability (a measure of sequence degeneracy throughout the region) of the position. The pileup string can be visually or automatically inspected to identify biases in the variant strandedness or determine whether the variant is 5’- or 3’-biased within the supporting reads. Low theoretical mappability can be used to determine whether reads aligned to certain positions could have been mapped confidently or whether they were likely to have been mismapped. AnnoVar [19] is used to determine a variant’s effect on both a conservative (RefSeq) and inclusive (UCSC) gene model set. This assessment also describes whether a variant changes an amino acid residue, whether it creates or removes a stop-codon, and the variant’s location: near an intron-exon boundary, within an intron, within a known non-coding RNA, or in an intergenic region. The annotation also includes five algorithms that predict the deleterious nature of nonsynonymous variants [21]. Variants are additionally annotated according to their frequency and presence in multiple variant collections (e.g., dbSNP, Thousand Genomes). Lastly, variants are annotated based on functional data for the gene in which they occur. These data include the known function of the gene (e.g., Swiss-Prot), any previous association of the gene with a disease (e.g., OMIM), post-translational modifications of the gene, and the expression profile of the gene across human organs. Together, these data are applied to assess the variant quality, its minor allele frequency, and any disease association while also elucidating the potential effects of the variant within a genetic framework.

### Metadata and LIMS

*Mercury* integrates external metadata resources and inputs such as a reference genome, sequence data locations, and a capture design bed file and therefore requires an integrated LIMS within the pipeline. Our LIMS solution is partitioned into three major modules: project management (PM), sample tracking (ST), and reporting. The PM module provides tools to define the purpose of the project and aggregate samples together. Such aggregation allows project-level decisions (e.g., capture design parameters, reference genome for mapping) to be applied to all relevant samples at once. The ST module provides features for tracking samples as they move through the sequencing center pipeline. Samples are tracked via a barcode given to each sample when it is accepted by the sequencing center. Once that barcode is recorded, all lab experiments and informatics analyses performed on the sample track this barcode. The barcode-based LIMS reporting module lets users monitor a sample’s progress in the pipeline and adjust the steps if necessary. The ability to see the history of the samples and sample data grouped or individually, is necessary for troubleshooting problematic samples and for developing and monitoring timelines.

To make *Mercury* portable, we provide communication “hooks” for transferring data between *Mercury* and LIMS. These hooks are scripts that can be modified to query or deliver data to any metadata resource. Examples of information served to *Mercury* from LIMS are the reference genome and previously generated SNP array data for quality assurance purposes. By decoupling *Mercury* from the sample tracking data we have built a more portable and compliance-ready pipeline, thus providing increased flexibility.

### Quality assurance, quality control, and error handling

In local environments, *Mercury* maintains an off-the-shelf validated open-source pipeline. Such transparency can pose challenges to the strict regulatory requirements that govern clinical sequence analysis. To support analysis best practices, we provide a set of documents, data, and validation tools (detailed manuals available with the code at https://www.hgsc.bcm.edu/content/mercury) as well as publically available test data [22]. Strict version control is maintained.

The *Mercury* pipeline generates a variety of performance metrics, including the number of pass-filter bases, read mapping fractions, concordance with orthogonal array genotypes, novel SNP rates, and transition-to-transversion ratios, which allow the user to gauge the quality of the final variant call results. Genotype arrays create a fingerprint of each sample upon intake that is then compared to the sequence data to validate sample identity. The governing principle of the process is to generate the quality control (QC) data as soon as possible and deliver that QC data into the LIMS (or other meta-data aggregating resource), but not to automatically interrupt the pipeline based only on QC data. The QC data are used in two ways. First, these data are considered in the clinical sign-out process for an individual run or sample. Second, the QC data can be used to detect systemic problems in the production pipeline, such as contaminated reagents or a malfunctioning sequencing instrument.

### Data security and compliance

The translational nature of modern genomic data and the scope of collaborations demand levels of data security unprecedented in our field. Based on our experience using *Mercury* on the DNAnexus platform to analyze thousands of samples, we have found a substantially higher security standard for data in the cloud than in most local systems. Managing the regulatory requirements of standards for handling sensitive medical data such as HIPAA, CLIA, dbGaP, and 21 CFR parts 11, 58, and 493 represents a considerable investment in compliance, security, and systems engineering expertise, and most local environments do not have the resources to maintain the required standards. By using cloud computing platforms, we are able to leverage diverse expertise, including modern, high-security data center technologies, best practices in encryption and authentication, and software system design to support clinical applications such as auditability, record retention and destruction, and reproducibility.

At the infrastructure level, DNAnexus uses data centers in high-security facilities with SAS-70/SSAE-16, PCI Level 1, and FISMA Moderate certifications. At the user level, it enforces best practices such as password strength and rotation, session expiration, and client encryption. All data access is carefully controlled, logged for auditing purposes, encrypted end-to-end (both in flight and at rest), integrity-verified, and replicated in at least three physically distinct data centers to ensure against loss. Data analysis is constrained to computing nodes that are sandboxed using virtualization and encryption technologies, and are versioned to ensure reproducibility and the ability to track data provenance. The software has undergone multiple third-party audits, including penetration testing by security experts, and the overall system has been ISO 27001 certified an internationally recognized standard for secure data management processes.

## Abbreviations

AWS: Amazon web services; BAM: Binary alignment/map; BWA: Burrows-wheeler aligner; CFR: Code of federal regulations; CLIA: Clinical laboratory improvement amendments; COSMIC: Catalogue of somatic mutations in cancer; dbGaP: The database of Genotypes and Phenotypes; EC2: Amazon elastic compute cloud; FISMA: Federal information security management act; Gbp: Giga base-pairs; HGMD: Human gene mutation database; HIPPA: Health insurance portability and accountability act; Indel: Insertion/Deletion; LIMS: Laboratory information management system; NGS: Next generation sequencing; NHGRI: National human genome research institute; OMIM: Online mendelian inheritance in man; PM: Project management; QC: Quality control; S3: Amazon simple storage service; SNP: Single nucleotide polymorphism; ST: Sample tracking; VCF: Variant call format; XML: Extensible markup language.

## Competing interests

JGR is a paid consultant of LaserGen. DNAnexus and AWS derive revenue from cloud computing services. The remaining authors declare that they have no competing interests.

## Authors’ contributions

JGR conceived and designed the study, developed software, performed data analysis, and participated in the drafting and writing of the manuscript. RAG and EB conceived and designed the study and participated in the drafting and writing of the manuscript. RD and GD conceived and designed the study. AC and NV developed software, performed data analysis, and contributed to the writing of the manuscript. MD and AS developed software and contributed to the writing of the manuscript. AE, MB, SW, and FY developed software. WS participated in the coordination of the study and contributed to the writing of the manuscript. CB and DMM participated in the coordination of the study. All authors read and approved the final manuscript.
